# Vitamin D Deficiency in Patients With Vitiligo: A Cross-Sectional Study From Basrah, Iraq

**DOI:** 10.7759/cureus.20733

**Published:** 2021-12-27

**Authors:** Zainab Mahmmod, Dooha K Ismael

**Affiliations:** 1 Department of Medicine, College of Medicine-University of Basrah, Basrah, IRQ

**Keywords:** vitamin d, vitiligo, veti score, systemic autoimmune disease, skin disease

## Abstract

Background and objective

Cholecalciferol (vitamin D3) plays a physiological role in melanogenesis in human skin. Vitamin D3 deficiency has become a common complication encountered in daily clinical practice. Recently, there has been growing interest in the role of vitamin D3 in the pathogenesis of vitiligo and its relevance in the treatment of the same. We have also noticed an increase in the rate of vitiligo with an associated aggressive extension of the lesions. In light of this, we conducted this study to analyze the incidence of vitamin D deficiency in patients with vitiligo and explore the effect of this deficiency on disease extension and severity.

Materials and methods

This was a cross-sectional study involving 46 patients with vitiligo. The affected body surface area of the patients was assessed using the Vitiligo Extent Tensity Index (VETI) score.

Results

Most of the vitiligo patients had very low levels of vitamin D (p<0.05), and a majority of the vitiligo patients with low vitamin D levels were females; however, this difference between females and males was not statistically significant (p=0.642). There was no significant effect of vitamin D levels on VETI scores (p=0.184).

Conclusion

Based on our findings, patients with vitiligo have a high incidence of vitamin D deficiency, and this deficiency is more common among females than males.

## Introduction

Vitiligo is characterized by a complete loss of melanocytes from the interfollicular epidermis [1]. Low serum vitamin D levels are found in many autoimmune diseases like systemic lupus erythematosus, diabetes mellitus (DM), and rheumatoid arthritis [2,3]. Vitamin D is a hormone that is synthesized in the skin. The active form of vitamin D, 1,25-dihydroxyvitamin D3, is a hormone that regulates calcium and bone metabolism, controls cell proliferation and differentiation, while also carrying out immunoregulatory functions [4]. However, the cause of low vitamin D3 in patients with autoimmune diseases remains unknown [5].

A significant body of data suggests that vitamin D3 has a strong immunosuppressive activity and its low levels are associated with autoimmune conditions including vitiligo [6]. Vitamin D may affect both innate and adaptive immune responses through receptors in T and B lymphocytes, macrophages, and dendritic cells [4]. In addition, vitamin D3 increases tyrosinase activity and melanogenesis via a nuclear hormone receptor - the vitamin D receptor in melanocytes [7].

## Materials and methods

Study design

This was a cross-sectional case-control study conducted in the Dermatology and Rheumatology units at the Al Sader Teaching Hospital and at the private clinic of a dermatologist (Basrah, Iraq), from November 2019 till December 2020. Participants’ verbal consent was obtained for their inclusion in the study. This study was approved by the IRB at the College of Medicine at the University of Basrah (number 03040815-2021).

Sample selection

Forty-six patients with vitiligo were involved in the study. Eligible patients included in the study were women and men diagnosed to have vitiligo by a dermatologist. The exclusion criteria were as follows: patients with vitiligo currently on treatment or any photosensitive medications, patients with DM, thyroid disease, and pregnant and lactating women. A group comprising 33 volunteers not related to the patients and who attended the hospital and matched in age and gender participated in the study as a control group.

Data collection

Data collection was performed using a questionnaire. Demographic and clinical features were collected, and these features included age, gender, smoking status, and comorbidities. Blood samples were collected from both patients and controls to measure vitamin D levels. The interpretation of the value of vitamin D was performed by the rheumatologist and the subjects were classified into three groups based on this: deficient (<20 IU), insufficient (20-30 IU), and normal (>30 IU). Affected body surface area was measured using the Vitiligo Extent Tensity Index (VETI) score [8], which is a system that measures the extent of vitiligo by a numerical score and combines the analysis of the extent and severity of vitiligo.

Statistical analysis

Statistical analysis was performed using the SPSS Statistics software version 22 (IBM, Armonk, NY). The median and interquartile range (IQR) were provided for quantitative variables. A p-value <0.05 was considered statistically significant.

## Results

Table 1 presents the demographical features of all participants. The mean age was 29.21 ±13 years among controls and 24.24 ±12.28 years for cases. Most of the controls and cases were females. There were no significant differences between cases and controls except for the presence of comorbidities: all cases were free from other diseases apart from vitiligo.

**Table 1:** Demographical features of cases and controls DM: diabetes mellitus; HT: hypertension; SD: standard deviation

Variables		Control		Case		P-value
		N	%	N	%	
Gender	Female	24	72.7	32	69.6	0.481
	Male	9	27.3	14	30.4	
Age (years)	Mean ±SD	29.21 ±13.05		24.24 ±12.28		0.164
	<25	13	39.4	28	60.9	
	25-45	16	48.5	15	32.6	
	45-65	4	12.1	3	6.5	
Smoking	No	29	87.9	39	84.8	0.481
	Yes	4	12.1	7	15.2	
Comorbidity	DM	1	3	0	0	0.004
	HT	1	3	0	0	
	Others	4	12.2	0	0	
	No comorbidity	27	81.1	46	100	

Of note, 45% of controls had normal serum vitamin D levels, while only 6.5% of cases had normal levels (p<0.05). On the other hand, most of the cases had very low levels of vitamin D (76% had deficient vitamin D levels), as shown in Table 2. This difference was highly significant.

**Table 2:** Vitamin D levels in cases and controls SD: standard deviation

Vitamin D levels	Controls		Cases		P-value
	N	%	N	%	0.00
Deficient	11	33.3	35	76.1	
Insufficient	7	21.2	8	17.4	
Normal	15	45.5	3	6.5	
Total	33	100.0	46	100.0	
Mean ±SD	28 ±15.99		16.16 ±11.18		

**Figure 1:**
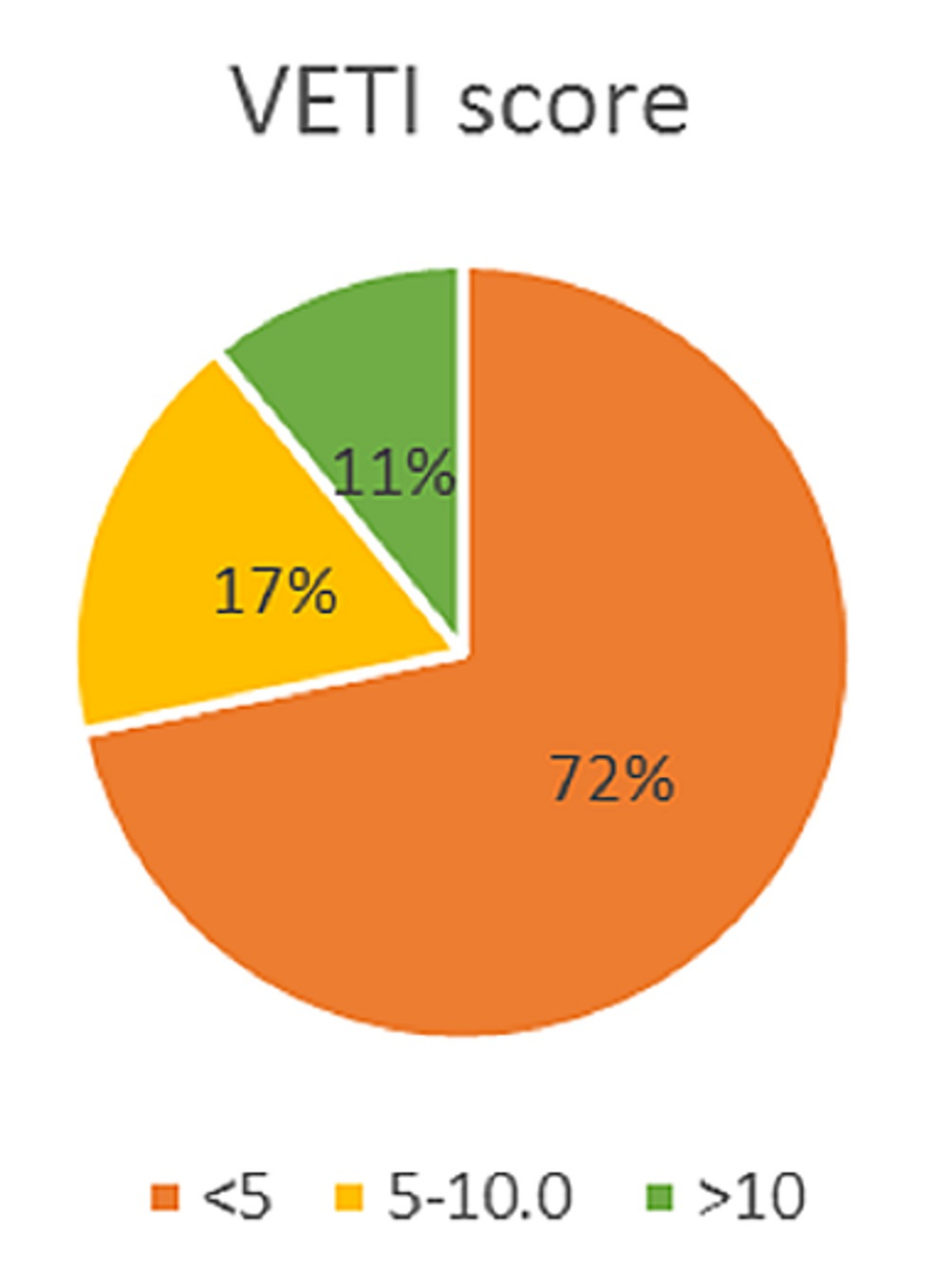
Percentage distribution of VETI score among patients VETI: Vitiligo Extent Tensity Index

Most patients with vitiligo were diagnosed early, and hence they had low VETI scores, as represented in Table 3 and Figure 1. The mean score was 4 ±3.7.

**Table 3:** VETI score among patients SD: standard deviation: VETI: Vitiligo Extent Tensity Index

VETI score	N	%
<5	33	71.7
5-10	8	17.4
>10	5	10.9
Total	46	100.0
Mean ±SD	4 ±3.799	

As shown in Table 4, most of the vitiligo patients who had low vitamin D levels were females (68.6% in the deficient group and 62.5% in the insufficient group); however, this difference between females and males was not statistically significant (p=0.642). Also, as shown in Table 4, a low level of vitamin D was most common in younger age groups, and this difference was also statistically insignificant (p=0.314).

**Table 4:** Distribution of vitamin D deficiency among different age groups and between genders in patients with vitiligo

		Vitamin D levels						
Variables		Deficient		Insufficient		Normal		P-value
		N	%	N	%	N	%	
Gender	Female	24	68.6	5	62.5	3	100.0	0.642
	Male	11	31.4	3	37.5	0	0.0	
	Total	35	100.0	8	100.0	3	100.0	
Age (years)	<25	21	60	5	62.5	2	66.7	0.314
	25-45	12	34.3	3	37.5	0	0.0	
	46-65	2	5.7	0	0	1	33.3	
	Total	35	100.0	8	100.0	3	100.0	

**Figure 2:**
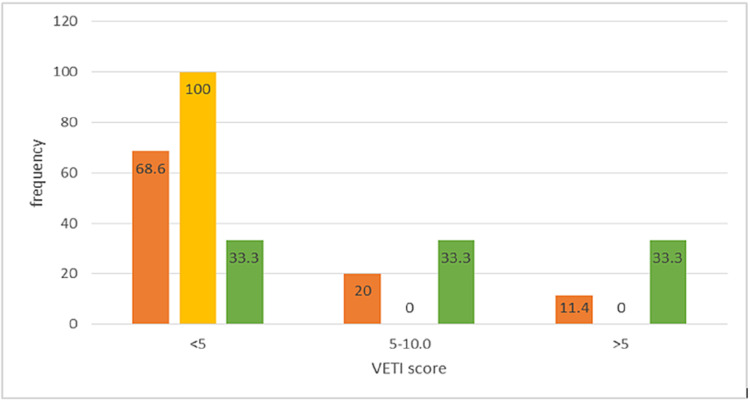
Relationship between vitamin D levels and VETI score VETI: Vitiligo Extent Tensity Index

As demonstrated in Table 5 and Figure 2, there was no significant effect of vitamin D levels on the VETI score (p=0.184).

**Table 5:** Relationship between vitamin D levels and VETI score VETI: Vitiligo Extent Tensity Index

	Vitamin D levels						P-value
VETI score	Deficient		Insufficient		Normal		
	N	%	N	%	N	%	
<5	24	68.6	8	100.0	1	33.3	0.184
5-10	7	20	0	0.0	1	33.3	
>10	4	11.4	0	0.0	1	33.3	
Total	35	100.0	8	100.0	3	100.0	

## Discussion

In this study, we found that most of the vitiligo patients had low vitamin D levels in comparison with the controls, and among a majority of them, the level was very low. This difference was statistically significant (p<0.05). Most of the patients with low vitamin D levels were females. This may be attributed to women wearing clothes that almost completely cover their body due to religious and cultural reasons, with only a small amount of the skin exposed (only face and hands), and weather conditions in our area (southern Iraq) that is characterized by long summers; moreover, most of the individuals were dark-skinned and very concerned about getting tanned that they avoid sunlight even indoors. VETI scores of most patients were low and did not directly correlate with vitamin D deficiency levels; this may be due to the fact that most of the included patients were incidentally newly diagnosed with early-onset disease. Most of the patients with low vitamin D were young, and this finding could be attributed to the fact that most patients in this study were adolescents and young adults and may also be related to a lifestyle trend among the youth in our society that has become widespread recently, which involves a reversed sleep cycle and waking up late.

The role of vitamin D in the pathogenesis of vitiligo is a subject of controversy, although many other studies have also found that vitamin D level is low in vitiligo patients, which is in line with our findings. Saleh et al. found significantly lower serum vitamin D levels in patients with vitiligo compared to healthy controls, thereby endorsing the use of vitamin D supplements in the treatment of vitiligo. However, they found that these deficiencies in vitamin D were not affected by age, which contrasts with the results of the current study. They also found that there was no correlation between vitamin D levels and affected body surface areas [9]. On the other hand, studies by Ustun et al. [10] and Karagüzel et al. [11] showed that there was no significant difference in serum vitamin D levels between patients and controls; they did not find any correlation of vitamin D deficiency either with age or affected surface areas. Moreover, Karagüzel et al. have reported that giving vitamin D supplements to patients with vitiligo who had low levels led to a decrease in lesion sizes from 66.1 ±58.3 cm^2^ to 48.0 ±52.6 cm^2^ after six months of treatment (p<0.001), in contrast to an increase of lesion size from 34.8 ±48.1 cm^2^ to 53.5 ±64.9 cm^2^ (p<0.01) in patients who received only topical therapy.

To the best of our knowledge, vitamin D significantly affects melanocytes and keratinocytes. Studies suggest that vitamin D3 increases tyrosinase activity and melanogenesis in vitro [12], which may lead to repigmentation in vitiligo skin lesions. Calcipotriol and tacalcitol, which are vitamin D analogs, are also known to induce repigmentation in patients with vitiligo [13,14]. Another study has indicated that vitamin D leads to immunomodulatory effects by inhibiting the expression of interleukin (IL)-6, IL-8, tumor necrosis factor (TNF)-α, and TNF-γ [15]. It has also been reported that the active form of vitamin D decreases the apoptotic activity in melanocytes that are induced by ultraviolet B (UVB) [16].

## Conclusions

Based on our findings, vitamin D levels were significantly low in patients with vitiligo in comparison with controls; however, we did not observe any significant effect of vitamin D on the occurrence and extension of vitiligo lesions. Further studies involving larger sample sizes and longer periods of time on different types of vitiligo with different degrees of severity are required to gain deeper insights into the topic. We recommend the use of vitamin D in the treatment of vitiligo, and further research is warranted to more comprehensively assess the efficacy of vitamin D in the treatment of vitiligo.
